# Comparative study of cavitands-based nanocapsule as a drug delivery vehicle for an anti-cancer and multiple sclerosis drug—A DFT study

**DOI:** 10.1098/rsos.250005

**Published:** 2025-09-10

**Authors:** Annum Ahsan, Sehrish Sarfaraz, Malai Haniti S. A. Hamid, Nadeem S. Sheikh, Khurshid Ayub

**Affiliations:** ^1^ Department of Chemistry, COMSATS University, Abbottabad Campus, KPK, 22060, Pakistan; ^2^ Chemical Sciences, Faculty of Science, Universiti Brunei Darussalam, Jalan Tungku Link, Gadong BE1410, Brunei Darussalam

**Keywords:** cavitand nanocapsule, benzimidazolone, ampyra, merceptopurine, drug delivery, quantum theory of atoms in molecules analysis

## Abstract

Nanoscale-assisted drug delivery systems give a platform to alter elementary properties associated with drug particles to limit their adverse effects. In this regard, deep benzimidazolone cavitand-based dimeric nanocapsule, which can act as good host for small guest molecules, is considered to be used as drug delivery vehicle. In the current study, we report the benzimidazolone cavitand-based nanocapsules as drug delivery systems for the drugs, i.e. ampyra (AM) and merceptopurine (MP) at M06−2x/6−31G(d,p) level of theory. AM and MP drugs interact with the nanocapsule with the interaction energies of −26.02 kcal mol^−1^ and −24.01 kcal mol^−1^, respectively. The results of quantum theory of atoms in molecules (QTAIM) and non-covalent index (NCI) analyses divulge that both the drug molecules are stabilized inside nanocapsule via the hydrogen bonding and van der Waals interactions. The transfer of charge is confirmed through electron density difference (EDD) analyses. Moreover, in the case of MP@cap slightly higher transfer of charge (natural bond orbital; NBO) is observed as compared with AM@cap. Furthermore, frontier molecular orbital (FMO) analyses show the higher energy gap reduction in the case of MP@cap as compared with nanocapsule. The FMO results are consistent with the results of interaction energies, NBO and EDD analyses. Additionally, we have employed *ab initio* molecular dynamics (AIMD) analysis to find the dynamical stability of drug delivery system after drug loading. Molecular docking has been performed for binding kinetics or the enzymatic interactions of the selected drugs. And, pH effect is studied for understanding the off-loading mechanism of the drugs, which clearly shows the decrease in E_int_ values pointing towards easier offloading. The analyses of values of dipole moment show that nanocapsule will carry MP drug more efficiently to the target site as compared with AM drug molecule. Overall, the results divulge that the benzimidazolone cavitand-based nanocapsule acts as better carrier for an anti-cancer drug molecule as compared with the other drugs.

## 1. Introduction

Drug ampyra (AM) is one of the three isomeric amines of pyridine with the molecular formula C_5_H_4_N–NH_2_. It is the first and only drug prescribed for the patients of multiple sclerosis (MS) who face walking difficulties [1,2]. AM works by blocking potassium channels present on nerve fibres’ surface. This leads to enhancement in the conduction of nerve signals in nerve fibres whose insulating myelin coating has been damaged by MS. Earlier, when AM was not presented, there had been no pharmacological treatment attainable for patients facing walking difficulty suffering from MS [3]. Despite the benefits associated with AM, like other drugs, it has some side effects as well like urinary tract infections, insomnia, dizziness, headache, nausea, back pain, impaired balance and so on. [1]. Moreover, it can also cause seizures and with the increase in dose, the risk of seizures also increases.

Another drug, merceptopurine (MP) is one of the well-known anti-cancer drugs [4]. It is mainly used for treatment of leukemia where it reduces the activity of the immune system. In cancer, the growth and multiplication of cells in the body becomes rapid. Anti-cancer drugs prevent or slow down the multiplication and growth process of these cells. These drugs work by targeting the genetic material inside the body cells. MP is one of the drugs used for chemotherapy. It also functions by stopping or reducing the cancer cells’ growth. Despite its significant therapeutic potential, its excessive intake can lead to a number of side effects on human body [5].

The drug delivery systems are one of the necessary inventions which help in avoiding various problems associated with drugs [6]. In this context, nanoscale drug delivery systems are essential. Nanoscale-assisted drug delivery systems give a platform to alter elementary properties associated with drug particles like their solubility, biocompatibility, half-life, and most importantly release characteristics [7]. They also help in the timed release of drug particles along with precision in drug targeting. Additionally, such drug carriers decrease the toxicity associated with drug molecules and allow efficient distribution of drug. Hence, a number of different nanomaterials that could work as carriers for drugs have been investigated [8]. Moreover, according to the literature, nanoparticles-based drug delivery systems that offer solutions to inferior absorption issue show noteworthy aptitude in treatment of cancer [9]. Examples of such nanoparticles include dendrimers, liposomes, polymeric micelles and so on. These nanoparticles have been applied as anti-cancer drug carriers and have shown effective results in minimizing side effects of drugs [10]. Similarly, amphiphilic copolymers containing self-assembled nanostructures act as anti-cancer drug carriers like doxorubicin [11]. Moreover, successful synthesis of large number of drug delivery systems which are responsive to near infrared radiations have also been reported. They show exceptional outcomes for the treatment of malignant cells. Yet another study (density functional theory (DFT) study) by Samanta *et al.* describes a drug delivery vehicle composed of fullerene (C_60_) surface for transport of temozolomide and carmustine [12]. In this study, they observed that the efficiency of off-loading process of drugs, i.e. temozolomide and carmustine, into biological systems is enhanced upon increasing the fullerene molecule’s polarity. The polarity of fullerene gets enhanced on adsorption of these drug on C_60_’s surface [12]. Furthermore, graphene and graphene oxide have also been studied as the nanomaterial drug carriers. Both act as potential candidates for delivery of different drugs. They show efficacious loading of drugs and targeted delivery along with their controlled release [13].

Efforts have been exerted in developing such novel targeted drug delivery systems but the targeted drug delivery systems still suffer from serious side effects and limited applicability due to drug instability, hydrophobicity and resistance [14,15]. In this regard, supramolecular approaches, including the cavitands, for example cyclodextrins, calix[n]arenes, pillararenes and cucurbiturils, have captured interest recently as promising substitutes in order to overcome these limitations [16,17]. Macrocycles can act as potential vehicles for various anti-cancer drugs by either self-assembly of macrocycles resulting into the formation of drug-loaded nanocapsules or host-guest complexation with anti-cancer drugs. This makes it feasible to use them in clinical research, because they offer many advantages. For example, development of host-guest complexes significantly increases the hydrophobic anti-cancer medications’ water solubility and bioavailability in physiological environments [14]. Moreover, the binding affinity of the host and guest molecules with each other can be changed by adjusting the conditions of the guest’s surrounding environment thus allowing better control over the release of the anti-cancer drug (i.e. guest) inside the cancer cells [14,16,17]. Supramolecular self-assembly could also improve chemotherapeutic agent’s targeting to cancer tissue resulting in improved anti-cancer activities along with reduced adverse effects [14].

Additionally, the literature related to recent advancements in supramolecular chemistry includes resorcinarene-based cavitands as they show exceptional encapsulation properties [18]. These cavitands were introduced for the first time [19] by Cram (in 1982). Later they were explored further in the form of cavitand-based nanoscale coordination cages [20], resorcinarene-based cavitands [21] and so on. Generally, the cavitand molecules are organic compounds which feature large concave cavities capable of making complexes with various compounds [18]. Lately, many supramolecular containers have been synthesized with the help of various non-covalent interactions [22].

**Figure 1.**
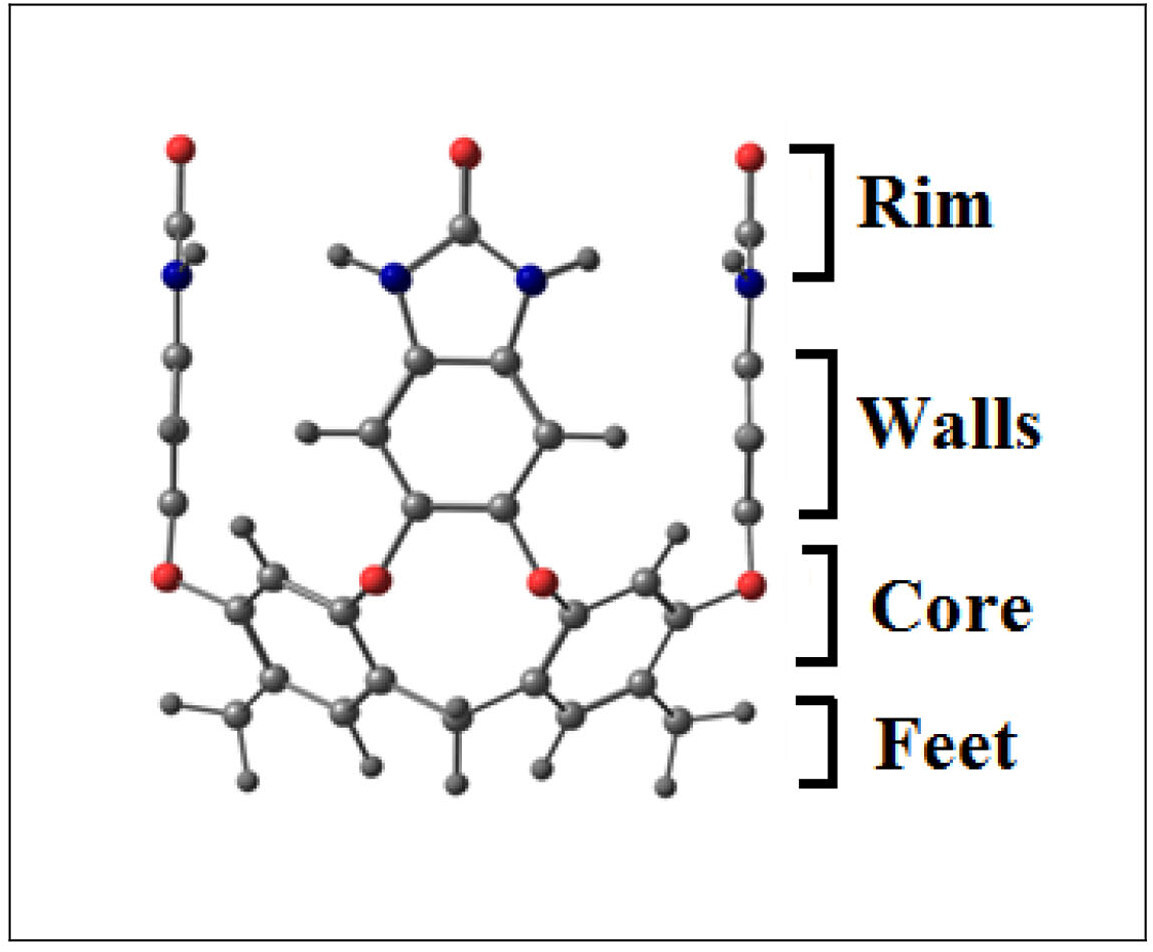
Resorcinarene-derived cavitand’s structure.

**Figure 2.**
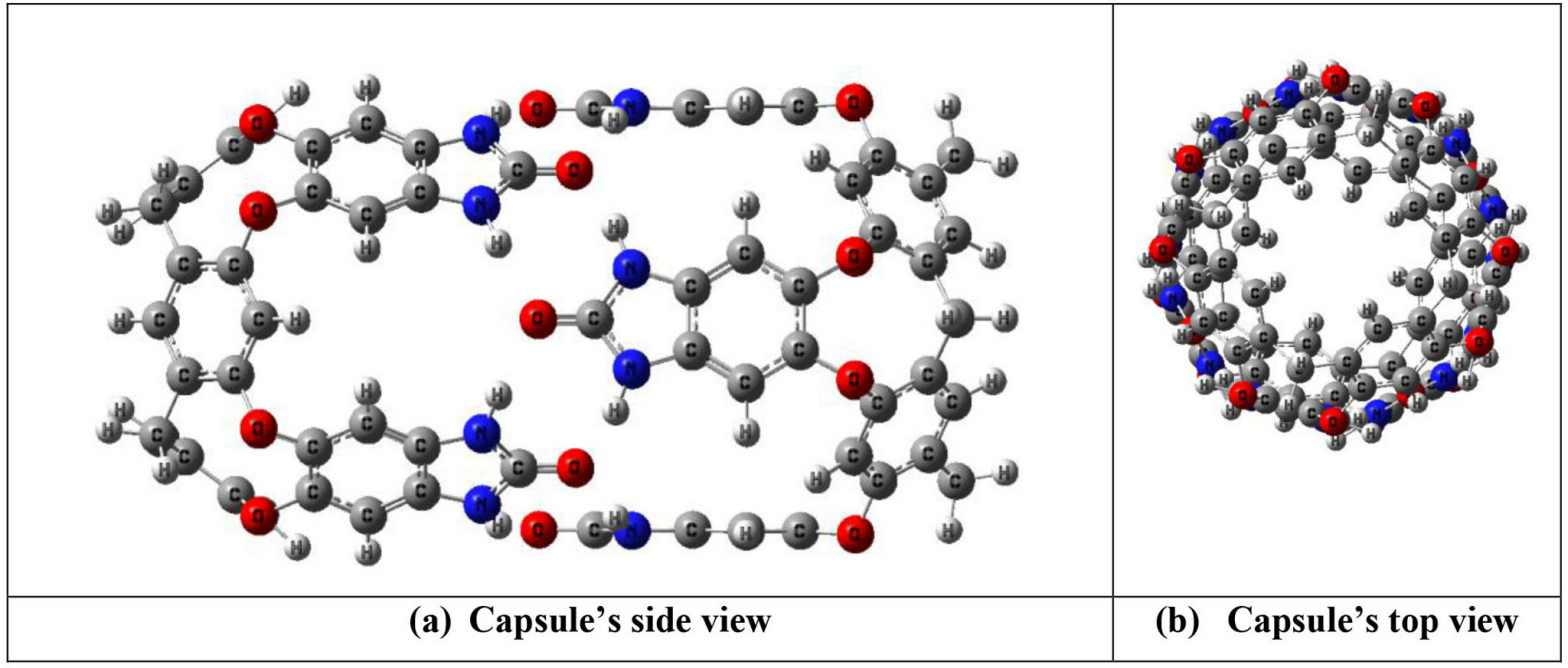
Geometry of deep benzimidazolone cavitand-based dimeric nanocapsule: (a) sideview and (b) top view.

Furthermore, dimeric nanocapsules based on deep benzimidazolone cavitands have also been reported [23]. They act as quite good encapsulating agents or hosts for small molecules. They are synthesized from a resorcinarene core through covalent bonding of walls, and the other features involved in stabilization of the shapes of these cavitands involve the rigidified walls, metal coordination, intramolecular hydrogen bonding patterns and also the solvent (water) [24,25] (figure 1). These cavitands further dimerize and give rise to dimeric nanocapsule. In the dimerization process, rim portion is involved, which allows intermolecular interactions to develop with the neighbouring cavitand molecule (figure 2). In the case of benzimidozolone cavitand rims, hydrogen bonding between the atoms at rim portion results in dimerization giving rise to nanocapsules, while groups present at the feet permit them to configure capsules in water. Other than hydrogen bonding, simple hydrophobic interactions can also form nanocapsules. Deep benzimidazolone cavitands show a number of uses in supramolecular chemistry, especially in selective recognition of molecules and sensors or molecular switches. However, the applications of these deep benzimidazolone cavitand-based dimeric nanocapsules as drug delivery systems have not been explored.

Additionally, we would prefer the nanocapsules over the other well-known drug carriers. In this regard, if we generally compare the well-known drug carriers like dendrimers, liposomes and polymeric micelles with nanocapsules. Liposomes and nanocapsules are both promising drug delivery systems, but they differ in structure, drug loading capabilities and stability. Liposomes are flexible, spherical vesicles with a lipid bilayer that can encapsulate both hydrophilic and hydrophobic drugs, offering versatility but potentially facing challenges with stability and drug loading efficiency [26,27]. Nanocapsules, on the other hand, are generally more rigid structures potentially offering better encapsulation abilities and control over drug release and targeting [28,29]. Moreover, nanocapsules are also considered superior to dendrimers and polymeric micelles as drug delivery vehicles due to their generally higher drug loading capacity, enhanced stability, and potential for targeted delivery [30].

In this respect, in the current study, we have used a dimeric nanocapsule (benzimidazolone cavitand-based) for designing a drug delivery system. The structural analysis reveals that the hydrogen bonding present between the cavitand molecules of a nanocapsule might improve the drug molecule’s off-loading at target site [31,32]. These nanocapsules have been studied for their excellent encapsulation abilities, but their role as a drug delivery vehicle is not explored. Despite all these characteristics of benzimidazolone cavitand-based nanocapsules, the major drawback associated with them is the cytotoxicity caused by benzimidazole-based molecules. The cytotoxicity of the benzimidazolone-based drug molecules can be reduced through functionalization of the benzimidazolone. Recently, a series of carboxamide-bearing benzimidazole derivatives have been reported. According to the study of their cytotoxicity profile, these compounds showed selective cytotoxicity or specificity towards the cancer cells and were divulged to be safe against human non-cancerous lung epithelial cells. Hence, through functionalization of the benzimidazolone its cytotoxicity can be addressed [33].

In the current study, we have reported for the first time the DFT-based theoretical examination on a dimeric nanocapsule composed of deep benzimidazolone cavitands as a proficient drug delivery system for AM and MP drugs. The nanocapsule’s unique features, i.e. electron-rich cavity and high surface area, allow the drug molecules to interact efficiently with it. We have compared the interaction of both the drugs, i.e. AM (used for treatment of MS) and MP (used as an anti-cancer drug) with the cavitand-based nanocapsule.

Additionally, the reasons behind choosing these two drugs specifically are AM’s short half-life (3–4 h) and MP’s narrow therapeutic window, which makes them strong candidates for nanocarrier optimization. A drug’s half-life is the time it takes for half of the drug to be eliminated from the body. A short half-life means the drug’s effects will fade quickly, requiring more frequent dosing. Nanocarriers can help extend the drug’s duration of action. A narrow window means the drug is more prone to toxicity at higher doses and less effective at lower doses. Nanocarriers can help improve the therapeutic window by allowing for more precise and controlled drug delivery. Nanocarriers can improve drug stability, increase solubility, and reduce the risk of side effects by targeting specific tissues or cells. Hence, a short half-life and narrow therapeutic window are reasons to use nanocarriers with AM and MP.

## 2. Computational methodology

In the current study, Gaussian 09 software package has been used for all the calculations [34] at M06−2x/6−31G(d,p) level of theory of DFT. For the purpose of visualization of structures, Chemcraft and GaussView 5.0 package are used [35,36]. During optimization, various orientations of drugs inside capsule are computed. The energies of all the orientations are analysed, and most stable geometry is selected among all (the geometry with lowest energy) and that stable geometry is used for further study. M06−2x functional is a global hybrid. It comprises 54% HF exchange and has been acknowledged as a functional that provides reasonable results for the complexes containing dispersion forces. In the literature, a number of examples with similar systems studied at the same level of theory have been reported [9]. Moreover, the confirmation of the true minima of the optimized orientations is done through the frequency analysis.

The interaction energies for each drug-capsule complex (i.e*.,* AM@cap and MP@cap) are evaluated by the following formula:


(2.1) $$\Delta E=E_{\text{complex}}-(E_{\text{drug}}+E_{\text{cap}}).$$

E_complex_, E_drug_ and E_cap_ denote the energies of complex, drug and capsule (bare), respectively.

Moreover, we have also calculated interaction energies at other dispersion-corrected functionals, i.e. ωB97X-D/6−31G(d,p) and B3LYP-D3/6−31G(d,p), for benchmarking and validation of the results calculated at M062X/6−31G(d,p). Additionally, as the whole study is conducted at M06−2X/6−31G(d,p), we have considered some higher basis sets with M06−2X for the validation of chosen method’s accuracy for our systems. The chosen basis sets are def-d2svp and def-d2tzvp.

Quantum chemical calculations which involve fragments interacting with each other are more likely to have basis set superposition error (BSSE). Therefore, corrections are required in such cases. Counterpoise method can be used to correct the energy through the following equation:


(2.2) $$\Delta E_{\text{int, CP}}=E_{\text{int}}–E_{\text{BSSE}}.$$

The solvent effects are critical in drug delivery studies as the drug will be delivered in the biological systems. In this respect, the calculations have also been performed in solvent, i.e. water, using the polarizable continuum model (PCM). The purpose of calculations in solvent is to study effect of presence of solvent on the stability of encapsulated drug molecules. The values of interaction energies in solvent are given in table 1.

**Table 1.** Interaction energies (E_int_ in kcal mol^−1^), BSSE corrected interaction energies (E_int(BSSE)_ in kcal mol^−1^), interaction energies in solvent (Eint(solvent) in kcal mol^−1^) energy of LUMO (E_L_ in eV), energy of HOMO (E_H_ in eV), HOMO-LUMO energy gap (E_g_ in eV) and NBO charges on drug (Q_drug_, |e|) in drug@cap complexes (drug = AM and MP).

drug@cap	E_int_	E_int(BSSE)_	E_int(solvent)_	E_L_	E_H_	H-L gap	NBO_(drug)_
cap	—	—	—	−0.25	−7.34	7.09	—
AM@cap	−24.01	−17.23	−21.48	−0.38	−7.04	6.66	−0.02
MP@cap	−26.02	−24.18	−19.63	−0.81	−6.80	5.99	−0.51

After the optimization, electronic properties have been explored through the analysis of frontier molecular orbitals (FMOs) i.e., the highest occupied molecular orbitals (HOMO) and the lowest unoccupied molecular orbitals (LUMO) [37], HOMO-LUMO gaps (H-L gaps) and natural bond orbitals (NBOs) at the same level of theory.


(2.3) $$E_{g}=E_{L}-E_{H}.$$

Moreover, non-covalent interactive forces among the drug-capsule complex are examined through quantum theory of atoms in molecules (QTAIM) analysis. QTAIM analysis describes the nature of interactions via the parameters, i.e. potential energy density V(r), electron charge density (*ρ*), energy density (H(r)), Laplacian of electron density (∇^2^*ρ*), Lagrangian kinetic energy G(r) and E_int_. Additionally, QTAIM analysis helps in calculating non-covalent interaction forces between the fragments (drug and capsule) through bond critical points (BCPs) [38–40]. Furthermore, electron density difference (EDD) analysis is employed for the purpose of exploration of electron-transfer behaviour between fragments. For the confirmation of electronic properties, density of states (DOS) spectra are generated through GaussSum software [41].

For having better acumen into the interaction forces between the drug molecules and capsule, non-covalent index (NCI) analysis is used. NCI helps to distinguish between the various types of interactions, i.e. electrostatic forces, van der Waals interactions and steric repulsions. NCI analysis provides relationship between reduced density gradient (RDG) and electron density (*ρ*) by the equation, i.e. [42],


(2.4) $$RDG=\frac{1}{2{\left(3\pi \right)}^{1/3}}\;\frac{\nabla \rho }{\rho^{3/4}}.$$

The three-dimensional plot provided through NCI analysis is used for the visual analysis of repulsive and attractive interactive forces. Three-dimensional isosurfaces show three colours, i.e. red, green and blue which determine the nature of various non-covalent interactions. Green colour characterizes weaker forces present in complexes among drug and capsule (London dispersion interactions), blue colour presents the strong interactive forces like hydrogen bonding in complexes, and the steric repulsive forces are shown by red colour. The graphs (two-dimensional) are attained through NCI by plotting RDG (a.u.) versus the product of density and the sign of second eigenvalue (*sign*(*λ*_2_)*ρ* (a.u.)). Moreover, colours in both the three-dimensional isosurfaces and two-dimensional graphs give the same information. The software used for generation of NCI plots is Multiwfn 3.8 [43].

Furthermore, the relation between recovery time and desorption energy of the encapsulated drug molecules has been studied. The recovery time can be theoretically calculated through transition state theory, by following equation:


(2.5) $$\tau =vo-1_{exp}(-E_{ads}/k_{BT})$$

In the current study, we have also employed *ab initio* molecular dynamics (AIMD) analysis to find the dynamical stability of drug delivery system after drug loading. The parameters considered to confirm the stability of selected systems are temperature, total time duration and time step. AIMD analysis simulations are carried out at 310 K (body temperature). The total time for each set of simulations is set at 1000 fs with time step of 6 ps.

Additionally, for studying the binding kinetics or the enzymatic interactions, the molecular docking studies have been performed in order to predict the interaction of protein (enzyme) with the drug molecules (ligands).

## 3. Results and discussion

### 3.1. Geometry optimization

A dimeric nanocapsule is used as a drug delivery system in the current theoretical study, which is based on deep benzimidazolone cavitand produced from resorcinarene core (figure 2) [44]. The deep cavitand contains benzimidazolone rim (figure 1), which is able to produce a dimeric-nanocapsule in water. The dimerization is attained through the interactions (mainly hydrogen bonding) between atoms at the rim portion of cavitands, i.e. hydrogen atoms and oxygen atoms. The nanocapsule shows hollow *C_4v_* symmetry with symmetrical vase conformation attained through intermolecular hydrogen bonding interactions between two adjacent benzimidazolone fragments [45]. The interaction distances for all hydrogen bonds existing between oxygen and hydrogen atoms present at the rim of cavitands are 1.89 Å.

**Figure 3.**
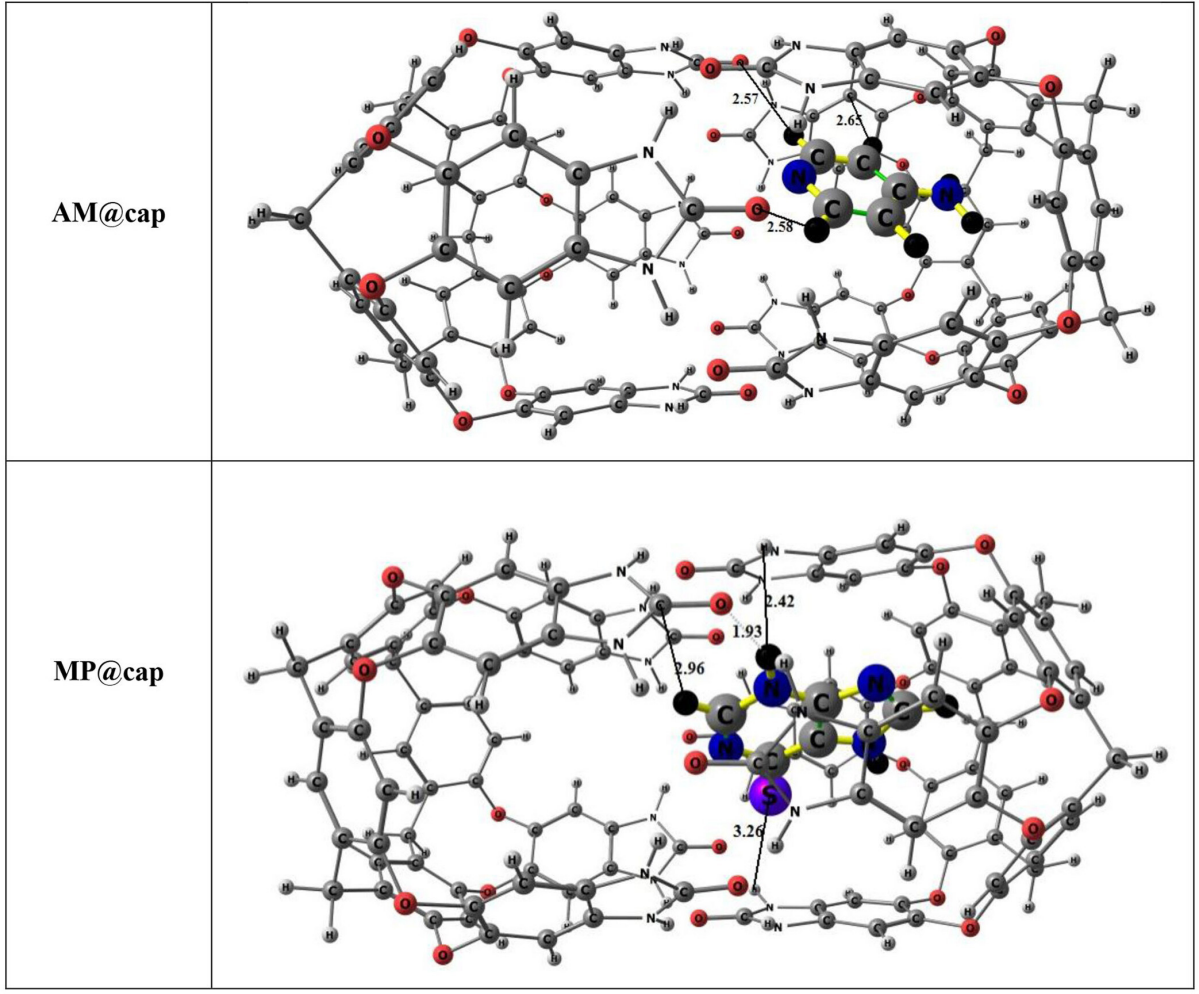
Optimized structures of drug-nanocapsule complexes, i.e. AM@cap and MP@cap (where AM = ampyra, MP = merceptopurine)

Concerning investigation of nanocapsule for drug delivery system, two drugs, namely AM and MP, have been selected for encapsulation. A number of possible orientations have been considered for both the drug molecules inside the capsule’s cavity in order to get the lowest energy or most stable configuration (electronic supplementary material, figure S1). The orientations differ from one another on the basis of position of drug inside. We have tried to study the interaction of the drugs with nanocapsule by considering every possible site inside the cavity. Then, the most stable geometries have been used for subsequent analysis and are shown in figure 3. The selection of most stable geometries is based on interaction energies mainly (calculated through equation (2.1)), i.e. the configuration which shows the lowest energy is considered as the stable one. Moreover, the frequency calculations have been performed to confirm the absence of imaginary modes. With all the positive vibrational frequencies, our structures are the true minima.

Additionally, interaction distances between atoms of drug molecules and nanocapsule are also a critical parameter. These distances help to quantify the strength of interaction between interacting fragments. The interaction distances and interaction energies are reported in table 1 for both AM@cap and MP@cap complexes.

For AM@cap complex, the configuration with the lowest energy −24.93 kcal mol^−1^ (i.e. the most stable one) is considered while for MP@cap complex, configuration with the lowest energy, i.e. −26.86 kcal mol^−1^ is considered. Interaction energy results show better results for MP encapsulation in nanocapsule as compared with AM. Moreover, the larger interaction energy for MP@cap can also be justified through the smaller interaction distances between the MP drug molecule and surrounding capsule atoms as compared with the distances shown by encapsulated AM molecule (figure 3).

The interaction energies show interaction strength between drug molecules and the drug carrier, i.e. nanocapsule. These E_int_ values show the affinity of the nanocapsule for holding the drug, as it is one of the important aspects while studying any system (carrier) for carrying a drug. The higher E_int_ for MP shows that the internal environment of the nanocapsule is more suitable for MP as compared with AM. Hence, the MP is more likely to stay inside the cavity while it will be carried to the reaction site as compared with AM. However, the E_int_ is not as high to affect or hinder the drug release at target site.

Additionally, in order to validate the chosen method’s accuracy for our systems we have considered ωB97X-D/6−31G(d,p), B3LYP-D3/6−31G(d,p), M06-def2svp and M06-def2tzvp for calculation of interaction energies (see table 2). The results of the calculations at all these selected pairs of functional and basis sets show that the encapsulation of both the drugs occurs through the exothermic interactions. Overall, with quite high negative E_int_ values at all these considered level of theories, the encapsulation of both the drugs show thermodynamic feasibility. However, the higher stabilization is shown by MP inside the nanocapsule’s cavity. Like the results calculated at M06−2X/6−31G(d,p), the results at other level of theories also show more stabilization of MP inside the cavity as compared with the AM.

**Table 2.** Interaction energies (E_int_ in kcal mol^−1^) of drug@cap complexes (drug = AM and MP) in calculated in solvent and at different methods.

drug@cap	E_int (solvent)_	E_int_ wB97XD	E_int_ B3LYP-D3	E_int_ M06-def2svp	E_int_ M06-def2tzvp
AM@cap	−21.48	−33.31	−34.82	−27.36	−21.68
MP@cap	−19.63	−34.69	−37.47	−32.39	−24.29

As ωB97X-D and B3LYP-D3 are well-known dispersion-corrected functionals, so the consistent results divulge the accuracy of the chosen method (M06−2X/6−31G(d,p)) for drug delivery systems. Moreover, yet further validation is awarded to the chosen method as the results calculated at all these higher basis sets are also consistent with M06−2X/6−31G(d,p).

The solvent effects are critical in drug delivery studies as the drug will be delivered in the biological systems. In this respect, the calculations have also been performed in solvent, i.e. water, using the PCM. The purpose of calculations in solvent is to study effect of presence of solvent on the stability of encapsulated drug molecules. The values of interaction energies in solvent are given in table 1.

For checking drug-nanocapsule systems’ stability in solvent, the interaction energies have been computed in the presence of the solvent (E_int(solvent)_). The values of E_int(solvent)_ are −21.48 and −19.63 kcal mol^−1^ for AM@cap and MP@cap, respectively. Although the values of E_int(solvent)_ are reduced as compared with E_int_ but overall negative values specify the stability of the drug@capsule systems in solvent as well. As the solvent intervenes between the atoms of drugs and nanocapsule, the charges get diluted and the overall interaction between the drug and nanocapsule reduces. However, the interaction is stronger enough even in the presence of the solvent showing the thermodynamic stability of the systems.

The BSSE-corrected interaction energies have also been computed through equation 2.2 (given in table 1). The values are −17.23 and −24.18 kcal mol^−1^ for AM@cap and MP@cap, respectively. The absolute values are definitely changed but the trend of BSSE-corrected energies matches with that of uncorrected energies. These values are also highest for encapsulation of MP in nanocapsule.

### 3.2. Electronic properties

#### 3.2.1. Analysis of frontier molecular orbitals

The analysis of FMO, i.e*.* HOMO and LUMO provides useful information regarding reactivity of a system. For the drug delivery systems, in order to predict the chemical reactivity of drug molecules towards the surfaces or vehicles (i.e. the nanocapsule in the current study), the energy gaps are the key factors. Any factor which results in reduction of E_g_ will result in an increase in reactivity of complexes.

**Figure 4.**
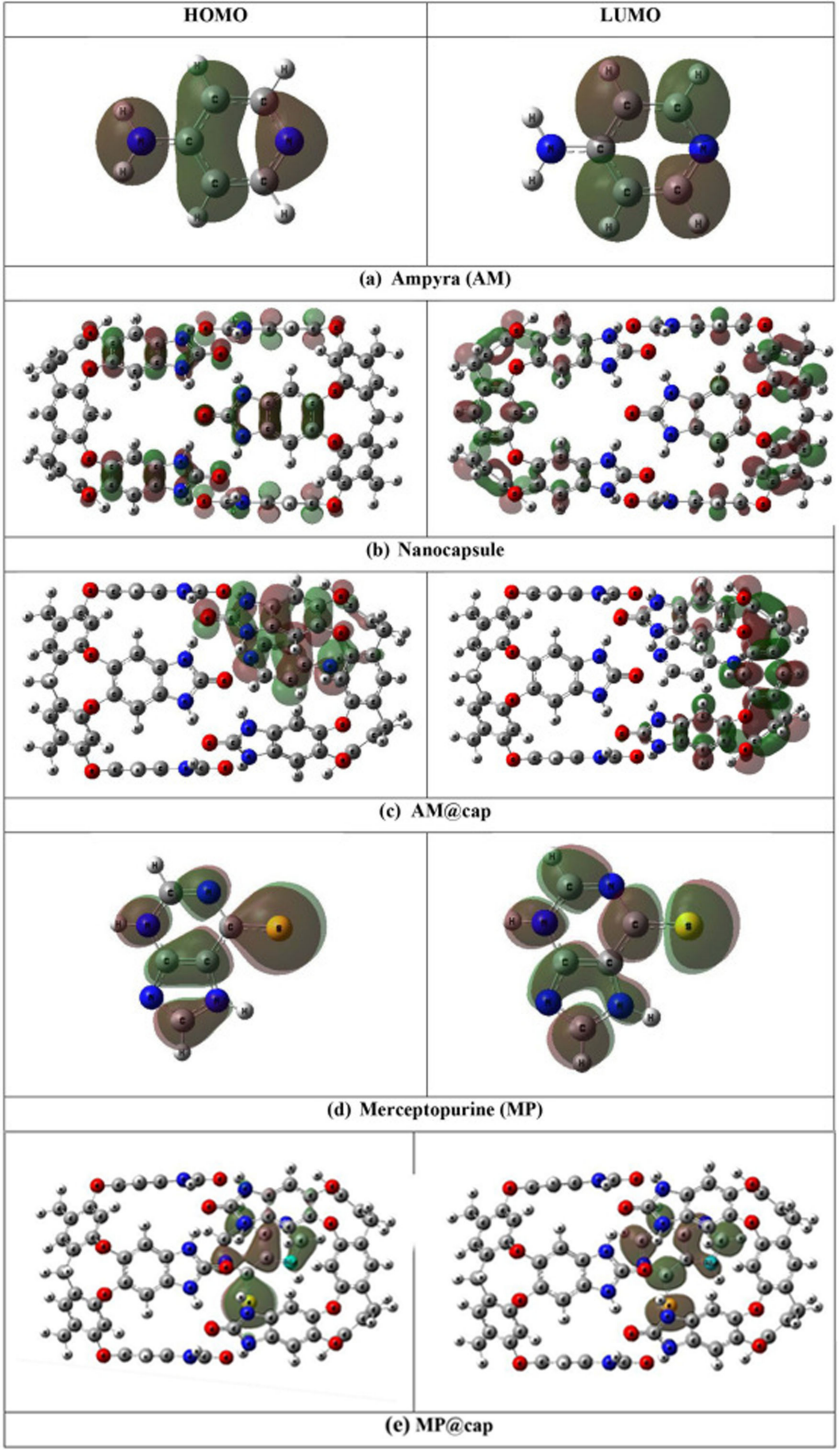
HOMO and LUMO of bare drug molecules, bare capsule and drugs@cap complexes.

Observing the orbital isosurfaces shown in figure 4, HOMO of nanocapsule lies over the rim part of nanocapsule, i.e. at all those aromatic rings of the nanocapsule which are surrounded by or which contain oxygen and nitrogen atoms, while LUMO can be seen mainly distributed over core part, i.e. over the aromatic rings of the nanocapsule which contain only carbon atoms. After the encapsulation of drug molecules, AM@cap complex shows the HOMO at AM molecule and that portion of cage which is interacting with the drug (AM), while MP@cap complex shows the HOMO localized completely over the drug (MP). On the other hand, LUMO of both the AM@cap and MP@cap lie over one side of the cage, i.e. the side near which drug molecules are present. There is a clear change in the position of FMOs after the drug and nanocapsule interact with each other. This points towards the better interaction between the fragments, i.e. drug molecules and nanocapsule.

On the other hand, analysing the energies of HOMO and LUMO, we see a clear decrease in HOMO-LUMO energy gap (E_g_) calculated through equation (2.3) after encapsulation of AM and MP into nanocapsule. In the case of AM encapsulation, the E_g_ decreases from 7.07 eV for bare capsule to 6.53 eV for AM@cap. The reduction in E_g_ is because of increase in energy of HOMO from −7.34 eV (for bare capsule) to −7.04 eV (for AM@cap) and decrease in energy of LUMO from −0.25 (for bare capsule) to −0.38 (for AM@cap). While after encapsulation of MP into capsule, the E_g_ decreases from 7.07 eV for bare capsule to 5.99 eV for MP@cap. The reduction in E_g_ is because of increase in energy of HOMO from −7.34 eV (for bare capsule) to −6.80 eV (for MP@cap) and decrease in energy of LUMO from −0.25 eV (for bare capsule) to −0.81 eV (for MP@cap). Hence, encapsulation results in lowering of E_g_ ultimately leading towards the increase in the reactivity of complex. Comparing the results for both the drugs, the change in E_g_ is higher for MP@cap as compared with AM@cap showing the nanocapsule to be a good carrier for carrying of MP drug, i.e. anti-cancer drug.

**Figure 5.**
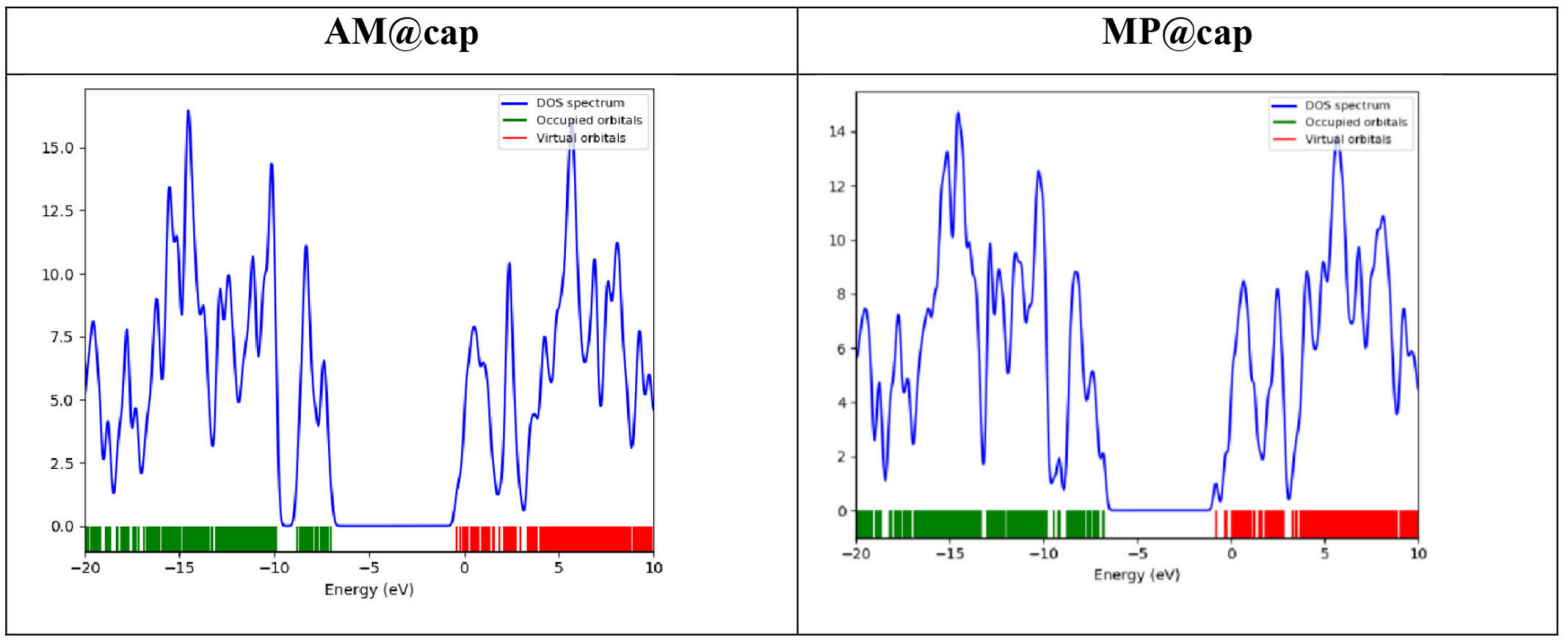
TDOS studies of the most stable drugs@cap complexes.

Moreover, the comparatively greater reduction in E_g_ for MP@cap justifies the greater strength of interaction between the drug (MP) and nanocapsule as well. The major reason can be MP’s more polar nature as compared with AM which definitely affects the drug–capsule interaction. The FMO analysis further highlights the stronger drug (MP) capsule interaction as merging of orbitals in this case is more efficient, which brings the HOMO and LUMO of the fragments closer, thus strengthening the interaction between the fragments. The values of energy of HOMO, LUMO and E_g_ are presented in table 1. The electronic properties are graphically represented by the total density of states (TDOS) graphs in figure 5. The graphs also confirm the energy of HOMO, LUMO and E_g_.

#### 3.2.2. Density of states analysis

The variations in electronic properties of the designed complexes (AM@cap and MP@cap) can further be verified through DOS analysis (figure 5). The DOS spectra for bare nanocapsule and the nanocapsule loaded with the drug molecules show clear differences. The peak shifts and changes in peak intensities show variation in the reactivity of the nanocapsule after encapsulation of drug molecules into it. After encapsulation of drug both the HOMO and LUMO are affected, which results in overall reduction in H-L gap. Moreover, the change in H-L gap higher for MP@cap complex as compared with AM@cap according to FMO analysis, is verified further through the DOS spectra.

#### 3.2.3. Natural bond orbital analyses

The NBO analysis on both the drug-capsule complexes is performed. NBO analysis helps to examine the charge that has been transferred between capsule and drugs. The results (table 1) show that there is negative charge (−0.01 |e|) on the drug (AM) inside nanocapsule indicating that the electronic charge has been shifted from nanocapsule towards the molecule of drug. In this case, N atoms of capsule lying near the drug show interaction with H atoms of drug molecules. In this way, electronic charge transfer takes place from capsule towards the AM drug. Observing the NBO charges on MP@cap complex, MP bears negative charge equal to −0.51 |e| inside nanocapsule thus revealing the charge transfer from nanocapsule towards the drug. Overall, the negative charge on the MP drug is greater than AM indicating that MP has interacted more strongly with nanocapsule as compared with the AM. The greater strength of interaction of MP with nanocapsule as compared with AM is also evident through the interaction energies calculated for them, i.e. greater for MP@cap complex than AM@cap.

#### 3.2.4. Electron density difference

**Figure 6.**
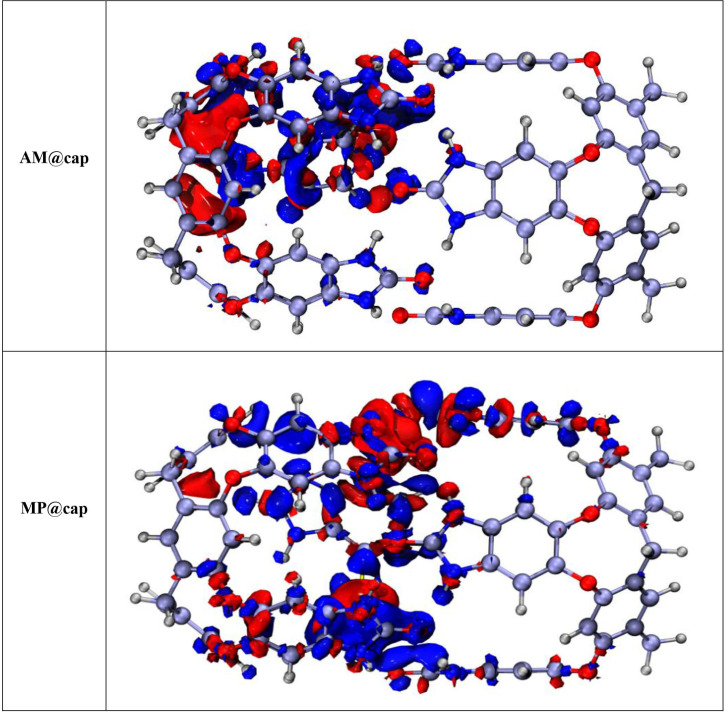
EDD plot of drugs@cap complexes: the electron density depletion is shown by red colour, while increase in electron density is shown by blue colour, (Isovalue = 0.004 a.u.).

Figure 6 presents the plots of AM@cap and MP@cap generated through EDD analysis. The isosurfaces of red colour depict the depletion of electron density while increase in electron density is represented by blue coloured isosurfaces. We can also say that the shift of electronic charges taking place between parts of drugs and nanocapsule which are interacting with each other are represented by red and blue coloured isosurfaces (*figure 6*). In AM@cap and MP@cap, the blue and red colours between the nanocapsule and drugs confirm the transfer of charge. Moreover, the prominent colour over the surface of drugs, i.e*.* AM and MP is blue, showing transfer of charge towards them. The quantitative charge transfer between drugs and nanocapsule has already been corroborated through the values of NBO charges.

## 4. Quantum theory of atoms in molecules analysis

For the purpose of examination of interactions, i.e. non-covalent, in drug@cap complexes (AM@cap and MP@cap), Bader’s QTAIM is used. For exploration of intermolecular interactions, it is considered as a best tool. QTAIM gives us BCPs which describe the nature of bonds with the help of various parameters. Among these parameters, Laplacian of charge density (∇^2^*ρ*(r)) is used to describe the non-bonding interactions’ nature and electron density (*ρ*) is used to evaluate the bond strength at BCP. Individual bond interaction energies (E_int_) also help in estimation of nature of non-bonding interactions. Espinosa approach is used to calculate the values of E_int_.


(4.1) $$E_{\text{int}}(a.u.)=1/2\;V(r).$$

Overall, E_int_ points toward hydrogen bonding when its value ranges from 3 to 10 kcal mol^−1^. Moreover, the values of Laplacian of electron density (∇^2^*ρ*(r)) less than zero (∇^2^*ρ*(r) < 0) specify the chemical bonding while, when greater than zero (∇^2^*ρ*(r) > 0) point towards weak intermolecular bonding interactions. Moreover, the formula used for calculating the energy density H(r) is


(4.2) G(r)+V(r)=H(r).

**Figure 7.**
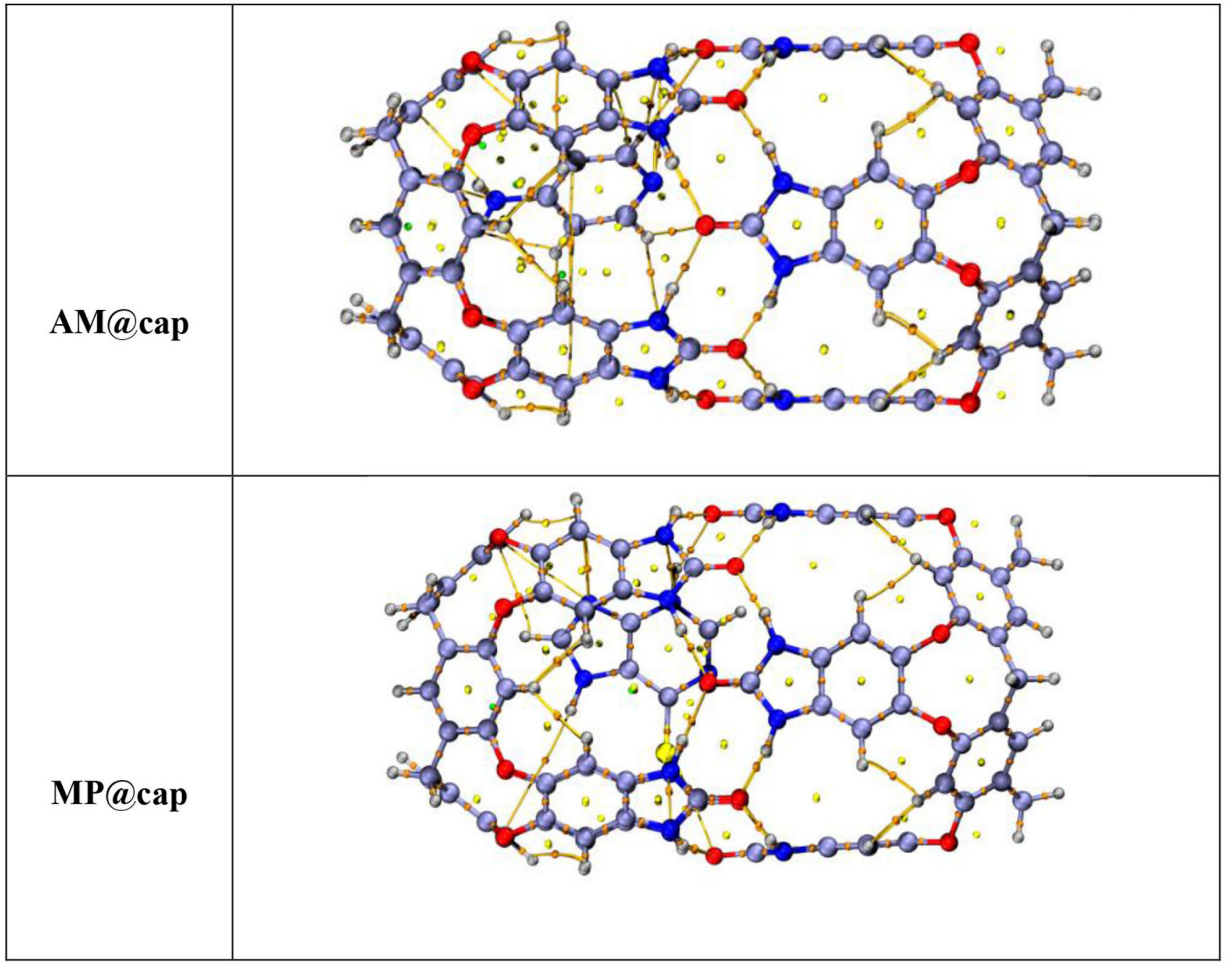
Bond critical points and bond paths in drugs@cap complexes, i.e. (*a*) AM@cap and (*b*) MP@cap obtained through QTAIM analysis. The lines present paths of bonds between drug and capsule, while coloured dots show BCPs.

The total energy density H_(r)_ less than zero (H_(r)_ < 0) and greater than zero (H_(r)_ > 0) reveal shared shell and closed-shell interactions, respectively. For further evaluation of intermolecular forces, -V(r)/G(r) (ratio) is calculated. The value of -V(r)/G(r) when calculated to be less than two shows weak bonding while when greater than two, it shows covalent bonding. Table 3 contains the values of above-mentioned topological parameters which are attained through QTAIM analysis of AM@cap and MP@cap. The BCPs among drug molecules and nanocapsule are presented in figure 7. A number of BCPs for both complexes can be seen indicating the existence of various interactions among the atoms of drugs and nanocapsule. Eight BCPs can be seen in the stable geometry of AM@cap complex, i.e. two **H—N**, one **H—O**, two **N—N** bond, one **N—C** and two **H—C** interactions. The values of electronic density *ρ*(r) for AM@cap complex vary between 0.002 to 0.009 a.u. The values for Laplacian of electron density ∇^2^*ρ*(r) vary between 0.014 to 0.026 a.u. Moreover, values of H(r) calculated for all BCPs are positive. Additionally, E_int_ vary between −0.66 to −1.66 kcal mol^−1^. Overall results of QTAIM analysis obtained for AM@cap point towards weak van der Waals interactions present between encapsulated drug and capsule.

**Table 3.** Results of QTAIM analysis with different topological parameters, Laplacian (∇^2^*ρ*), electron charge density (*ρ*), potential energy density V(r), Lagrangian kinetic energy G(r), energy density (H(r)), and interaction density (E_int_).

drug@cap	drug@Cap	ρ (a.u)	∇^2^ρ (a.u)	G (r) (a.u)	V (r) (a.u)	H (r) (a.u)	-V/G	E_int_ (kcal mol^−1^)
	**H226---N161**	0.006	0.019	0.004	−0.0035	0.0006	0.86	−1.09
	**H227---N173**	0.002	0.020	0.004	−0.0038	0.0006	0.95	−1.19
	**H227--- O107**	0.007	0.026	0.005	−0.0052	0.0006	1.04	−1.63
AM@cap	**H224---C136**	0.009	0.026	0.005	−0.0047	0.0009	0.94	−1.47
	**H224---C109**	0.004	0.014	0.002	−0.0021	0.0007	1.05	−0.66
	**N217---C97**	0.007	0.021	0.004	−0.0040	0.0006	1.00	−1.26
	**N218---N155**	0.008	0.025	0.005	−0.0053	0.0004	1.06	−1.66
	**N218---N154**	0.007	0.024	0.005	−0.0052	0.0005	1.04	−1.63
	**H227---C159**	0.007	0.029	0.005	−0.0036	0.0018	2.00	−1.13
	**H229---C109**	0.005	0.019	0.003	−0.0027	0.0010	0.90	−0.85
MP@cap	**N220---C131**	0.008	0.024	0.005	−0.0046	0.0008	0.92	−1.44
	**H228---O199**	0.025	0.080	0.020	−0.0210	0.0005	1.05	−6.59
	**N219---N161**	0.008	0.028	0.006	−0.0053	0.0008	0.88	−1.66
	**N221---O197**	0.001	0.917	0.001	−0.0011	0.0005	1.10	−0.35
	**S217---O195**	0.006	0.022	0.004	−0.0040	0.0008	1.00	−1.26
	**S217---N179**	0.008	0.027	0.006	−0.0054	0.0007	0.90	−1.69

^a^ a.u is for atomic units

For the other drug-capsule complex, i.e. MP@cap, eight BCPs, i.e. two **H—N**, one **N—C,** one **H—O**, one **N—N** bond, one **N—O,** one **S—O** and one **S—N** interactions are detected. In this case, the *ρ*(r) and ∇^2^*ρ*(r) are in the range of 0.001 to 0.025 a.u. and 0.019 to 0.917 a.u, respectively (overall greater than AM@cap). While interaction energies range from −0.35 to −6.59, the values point towards the existence of van der Waals forces at most of the interacting sites between drug (MP) and capsule, but between H228 and O199 there exist hydrogen bonding interactions with E_int_ of −6.59 kcal mol^−1^. Comparing both the complexes, QTAIM analysis divulges strong interaction forces between drug and capsule in the case of MP@cap complex as compared with AM@cap.

## 5. Non-covalent interactions analysis

**Figure 8.**
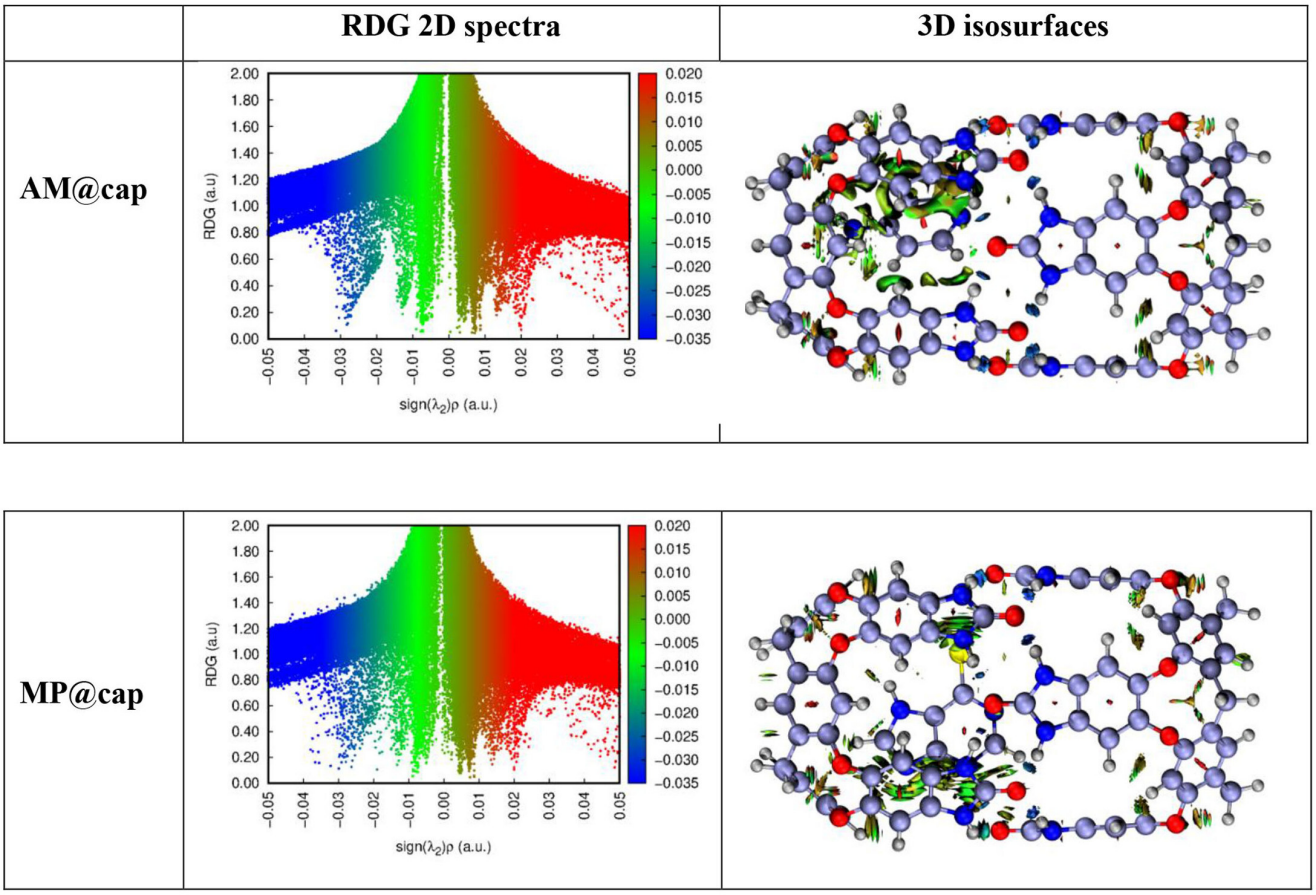
Three-dimensional isosurfaces and RDG two-dimensional spectra of drugs@capsule complexes (AM@cap and MP@cap) obtained via NCI analysis (isovalue = 0.5 a.u.). Strong electrostatic interactions are shown by blue colour, van der Waal’s forces are shown by green colour and repulsive forces are shown by red colour.

NCI analysis gives further acumen into the type of non-covalent interactions that are present between drug molecules and capsule. For the studied drug@cap complexes, the three-dimensional isosurfaces and two-dimensional NCI graphs are generated (shown in figure 8) in order to determine the repulsive and attractive forces’ nature between drugs and capsule. The three-dimensional isosurfaces show three colours, i.e. red, green and blue which help in differentiation of different non-covalent interactions (figure 8). Green colour characterizes weaker forces present in complexes among drug and capsule (i.e. London dispersion interactions), blue colour presents the strong interactive forces like hydrogen bonding in complexes [46] and the steric repulsive forces are shown by red colour. Here, during the generation of three-dimensional isosurfaces, the isovalue of 0.5 a.u. is used. Moreover, the strength of non-bonding interactions in complexes is directly related to the thickness of coloured patches in isosurfaces. The thicker and stippled patches show interactions of strong and weak nature, respectively.

The three-dimensional topologies for AM@cap and MP@cap are given in figure 8. There are observed patches of all the three colours between drug and capsule atoms. The coloured patches show that drugs are encapsulated inside the capsule’s cavity through van der Waals forces of attraction. Moreover, the patches surrounding the drug molecules inside nanocapsule possess the varying thickness. The non-uniformity in thickness around drug molecules shows that these molecules are interacting with nanocapsule through different energies. Additionally, we observe that in drug@cap complexes, light brown and green patches are not existing precisely between any two atoms. Rather the patches can be seen dispersed among a number of atoms. Conversely, the patches of blue colour can be seen precisely between two atoms, i.e. between an atom of drug molecule and an atom of nanocapsule. This shows that at some specific positions, hydrogen bonding is involved in encapsulation of drugs particularly between hydrogen and oxygen atoms of the interacting bodies. The results displayed in three-dimensional structures can be correlated to two-dimensional RDG plots. The spectra of complexes show spikes of bluish colour at −0.03 a.u. and green spikes are shown from 0.01 to −0.02 a.u. These spikes confirm that the encapsulation of drugs inside capsule are stabilized with the help of hydrogen bonding and London dispersion forces. Overall, NCI analysis shows that stabilization of drug molecules inside capsule is mainly assisted by van der Waals forces. Additionally, the off-loading of molecules of drug might also be assisted by these van der Waals interactions at the target site.

## 6. *Ab initio* molecular dynamics analysis

**Figure 9.**
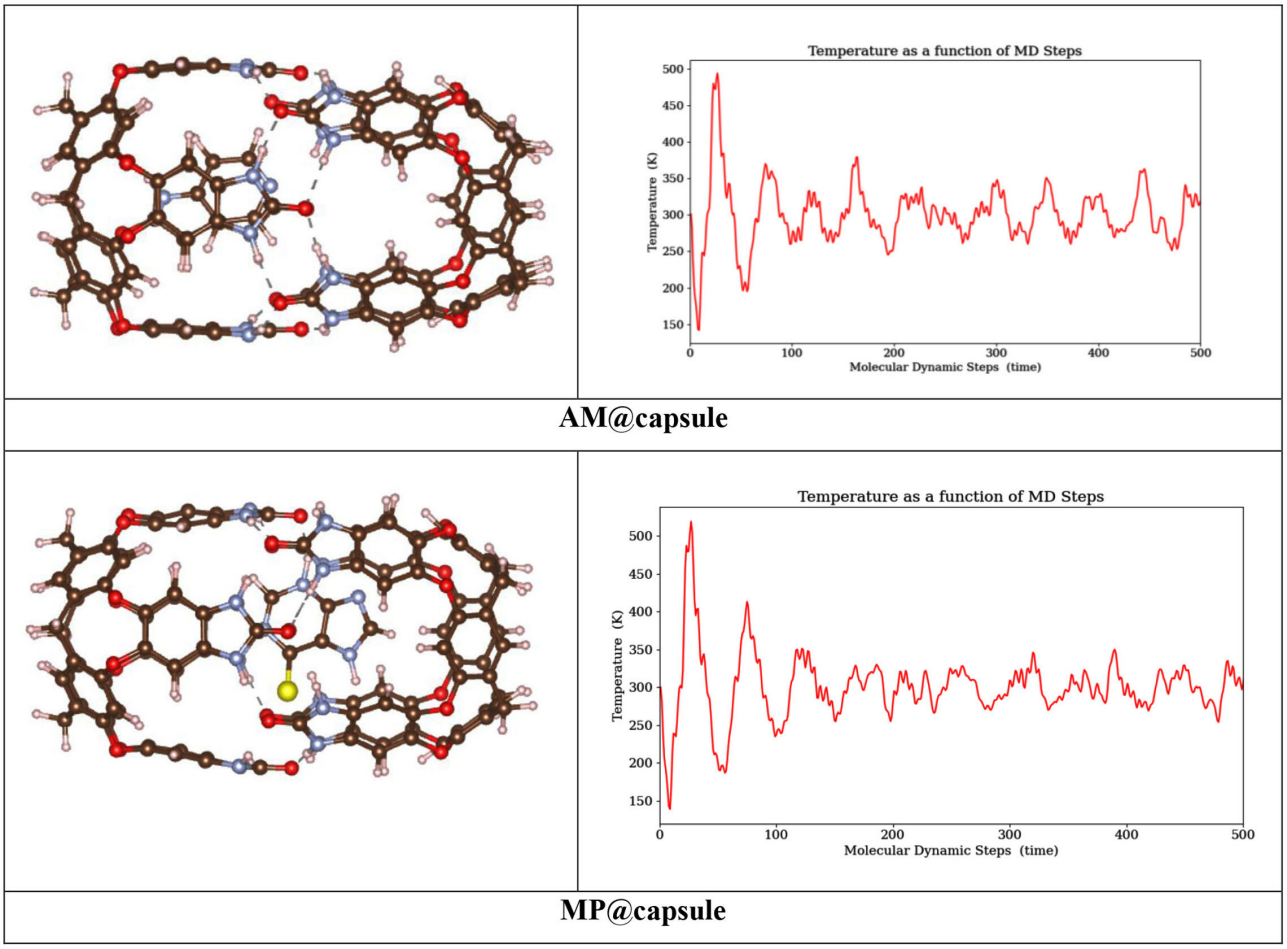
Results of AIMD analysis.

The dynamical stability of drug delivery systems before and after drug loading is analysed by AIMD analysis. The parameters considered to confirm the stability of selected systems are temperature, total time duration and time step. AIMD analysis simulations are carried out at 310 K (body temperature). The total time for each set of simulations is set at 1000 fs with time step of 6 ps. The geometry and their relative plots obtained through AIMD analysis are presented in figure 9. The topology of capsule resulted by AIMD analysis is showing negligible distortion. In addition, root means square deviation (RMSD) of capsule is plotted between energy (eV) and time (fs), which also shows little fluctuation. These findings show that capsules are suitable for drug delivery at body temperature (310 K). Geometries of AM@capsule and MP@capsule complexes analysed through AIMD analysis are almost comparable to geometry of capsule before drug loading. Moreover, the temperature time graphs of the complexes show that MP@capsule complex shows more stability as compared with the AM@capsule complex. The structure of MP@capsule shows fewer fluctuations after almost 100 MD steps as compared with AM@capsule. Furthermore, the related free energy graphs obtained through AIMD analysis (electronic supplementary material, figure S2) also show more stability of the MP@capsule as compared with AM@capsule complex. This shows that cavitand capsule stabilizes an anti-cancer more efficiently and can carry an anti-cancerous drug to its target site more proficiently as compared with the other drug.

### 6.1. Drug release studies

#### 6.1.1. Dipole moment

The value of dipole moment of the optimized bare capsule is zero. After formation of the complex with AM and MP, i.e. AM@cap and MP@cap, we see an increase in dipole moment up to 3.15 and 5.15 D, respectively. The reason behind increase of dipole moment is an overall change in symmetry of the nanocapsule after interaction with AM and MP. This interaction gives rise to new dipoles in complexes, i.e. at the electron donating and electron withdrawing portions. The dipole moment increase for complexes is useful for the purpose of their solubility in polar solvents (mainly water). These features point toward increase in hydrophilicity after complex formation, which will ultimately affect the movement of the drugs inside living systems.

Moreover, the calculated dipole moment for MP@cap is higher than that for AM@cap. The greater dipole moment is associated with higher value of the interaction energy as well. This is because the higher dipole moment demonstrates greater displacement in the distribution of electronic clouds. It impacts the binding affinity of the drug with nanocapsule and stability of drug-carrier complexes. This stability is important for ensuring that the drug remains bound to the carrier until it reaches the target site and is released effectively. Additionally, the higher dipole moment after complex formation of nanocapsule with MP will help to improve the complex’s (cap-MP) solubility in polar solvents, which is necessary for drug delivery in biological systems.

## 7. Recovery time

The relation between recovery time and desorption energy of the encapsulated drug molecules has been studied. Generally, the strong interaction between the species results in a long recovery time suggesting that the process of desorption will be difficult. The recovery time can be theoretically calculated through transition state theory, by equation 2.5.

According to equation 2.5, the more negative the values of E_int_, the more prolonged will be the recovery time. In order to calculate the desorption energy (E_d_), the activation energy (E_a_) must be overcome. In this case, E_a_ is equal to the interaction energy. Another way of prediction of E_d_ is taking -E_a_. So, the more negative the adsorption or interaction energy, the higher will be desorption energy and ultimately it will take longer recovery time in release of drug from nanocapsule.

We have calculated the recovery time (given in table 4) for both the drug molecules at three different temperatures, i.e. 298, 350 and 400 K. At 298 K, the recovery time of AM and MP has been calculated to be 3.9 × 10^3^ and 1.2 × 10^5^ s with the energies of desorption equal to −24.01 and −26.02 kcal mol^−1^ (see table 4). It can be noticed that the MP with the higher E_int_ also shows the longer recovery time as compared with AM. Moreover, by increasing the temperature to 350 K, the recovery time is decreased to 9.7 and 1.7 × 10^2^ s for AM and MP, respectively. Yet further increase in temperature to 400 K decreases the recovery time to 0.12 and 1.62 s, respectively. The results show that with the increase in temperature, the recovery time decreases. The same relation between both the parameters is given by equation 2.5. Overall, results show that higher recovery time is shown by MP at all the temperatures. The recovery time results are also consistent with other results. The stronger interaction of MP with nanocapsule is also studied through the E_int_, NBO, EDD, NCI and QTAIM analyses. The stronger interaction of MP results in prolonged recovery time for MP.

**Table 4.** The calculated recovery time at different temperatures.

drugs	temp.	τ
AM	380	3.9 × 10^3^
AM	390	9.70
AM	400	0.12
MP	380	1.2 × 10^5^
MP	390	1.7 × 10^2^
MP	400	1.62

## 8. Molecular docking

For studying the binding kinetics or the enzymatic interactions the molecular docking studies have been performed in order to predict the interaction of protein (enzyme) with the drug molecules (ligands).

**Protein:** Kv1.2–Kvβ2 potassium channel complex

**Ligands:** ampyra (AM)

This research involves the investigation of interaction between the protein ‘Kv1.2–Kvβ2 potassium channel complex’ and ligand ‘ampyra’ by using molecular docking through PyRx software. The three-dimensional structure of the Kv1.2–Kvβ2 potassium channel complex (PDB ID: 2a79) has been downloaded from RCSB Protein Data Bank (PDB) database and structure of ligand has been downloaded from PubChem in SDF format. The protein structure is prepared by using Discovery studio and then docked by using PyRx software, after this the results have been visualized through Discovery studio.

### 8.1. Molecular docking results

The results of binding energy, ligand efficiency and inhibition constant of AM drug with Kv1.2–Kvβ2 potassium channel complex obtained after the molecular docking are given in table 5. The strength of the interaction between the drug molecule and the protein depends on the more negative value of binding energy. The low value of inhibition constant indicates that the drug is the potent inhibitor at this target site or protein.

**Table 5.** Binding energy, ligand efficiency and inhibition constant of ampyra with Kv1.2–Kvβ2 potassium channel complex.

ligand	protein	binding energy (kcal mol^−1^)	ligand efficiency (kcal mol^−1^)	inhibition constant (ki) (μM)
ampyra	Kv1.2–Kvβ2 potassium channel complex	−4.5	0.643	5.0250

**Figure 10.**
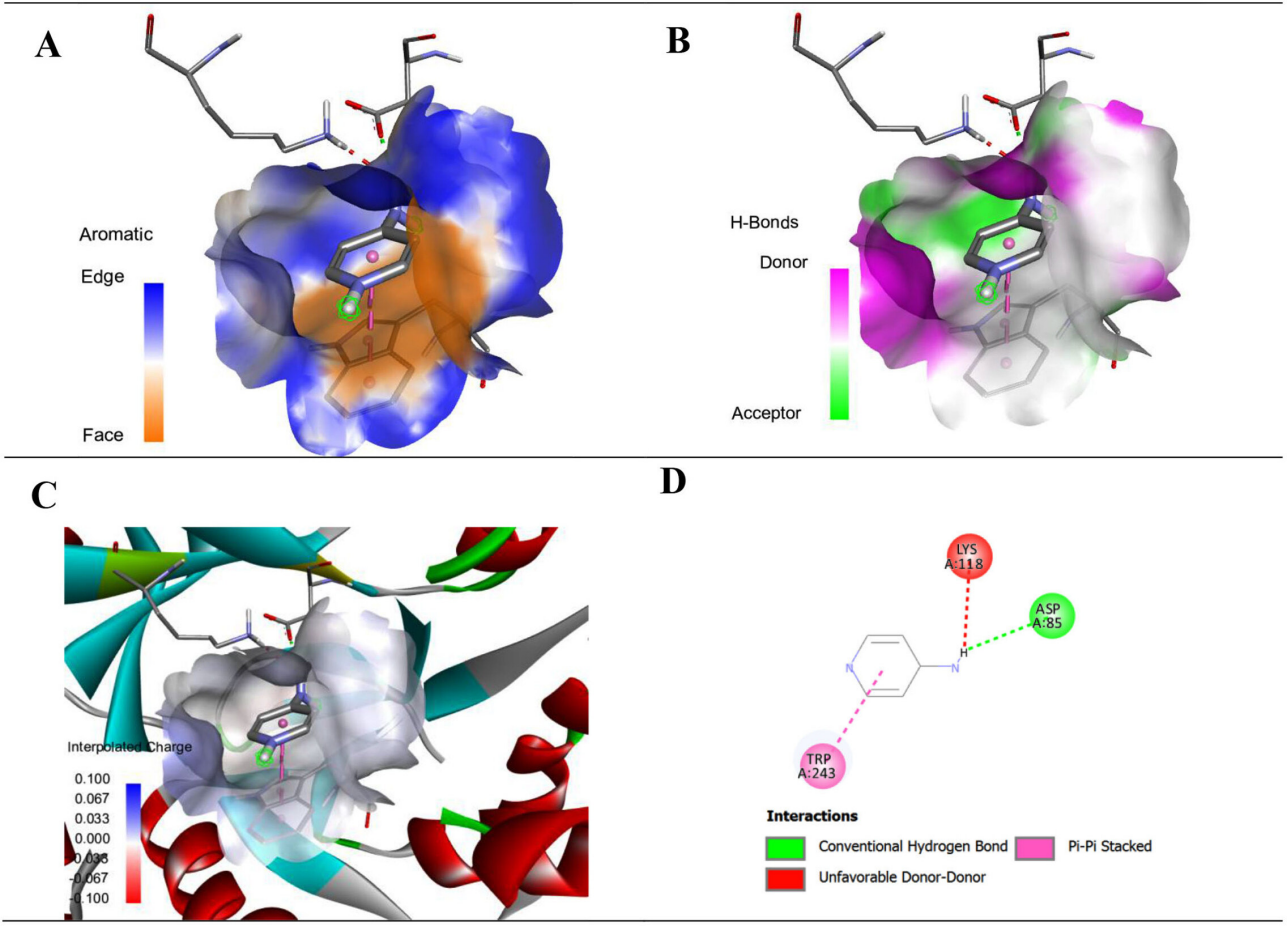
Aromatic (A), hydrogen bond (B), charge (C) and two-dimensional (D) interactions of the drug ampyra with the protein Kv1.2–Kvβ2 potassium channel complex PDB ID = 2a79

The detailed interactions between AM and Kv1.2–Kvβ2 potassium channel complex are given in table 6 with specific amino acid, and the corresponding distances between the interacting sites are also given in the same table. There are multiple bonds formed between drug (AM) and target such as conventional hydrogen bond and pi-pi stacked bonding ensuring strong binding between AM and Kv1.2–Kvβ2 potassium channel complex. The visual illustration of these interactions is given in figure 10.

**Table 6.** The binding interactions of ampyra with Kv1.2–Kvβ2 potassium channel complex.

ligand	amino acid	interactions	distance
ampyra	N:UNK1:H - A:ASP85:OD1	conventional hydrogen bond	2.16409
	A:TRP243 - N:UNK1	Pi-Pi stacked	3.94931
	A:TRP243 - N:UNK1	Pi-Pi stacked	3.99175

**Protein:** Human serum albumin (HSA) and hypoxanthine-guanine phosphoribosyltransferase (HGPRT)

**Ligand:** merceptopurine (MP)

The molecular docking has been performed via PyRx to investigate the interaction of MP drug with its target protein HSA and HGPRT. The three-dimensional structure of the MP is obtained from PubChem database in SDF format. The structures of targets (PDB ID: 1e78 and 4fm9) have been obtained from RCSB PDB database. The protein structure is prepared from Discovery studio by removing water and ligands attached, and hydrogens added. The targets were docked by MP drug using PyRx and resulting interactions are visualized in Discovery studio visualizer.

After docking process, the binding energies, ligand efficiencies and inhibition constant of the drug and target complex are given in table 7.

**Table 7.** The binding energy, ligand efficiency and inhibition constant of merceptopurine with the protein HSA and HGPRT.

ligand	protein	binding energy (kcal mol^−1^)	ligand efficiency (kcal mol^−1^)	inhibition constant (ki) (μM)
merceptopurine	(HSA) human serum albumin	−5.5	0.550	9.2909
	HGPRT	−5.3	0.530	1.3022

More negative binding energies show stronger interactions between the drug and protein. In the case of MP, with both the proteins, i.e. HSA and HGPRT, the binding energies are comparable and almost equal; however, the lower value of inhibition constant with HGPRT is observed showing that MP is a more potent inhibitor of HGPRT protein.

**Figure 11.**
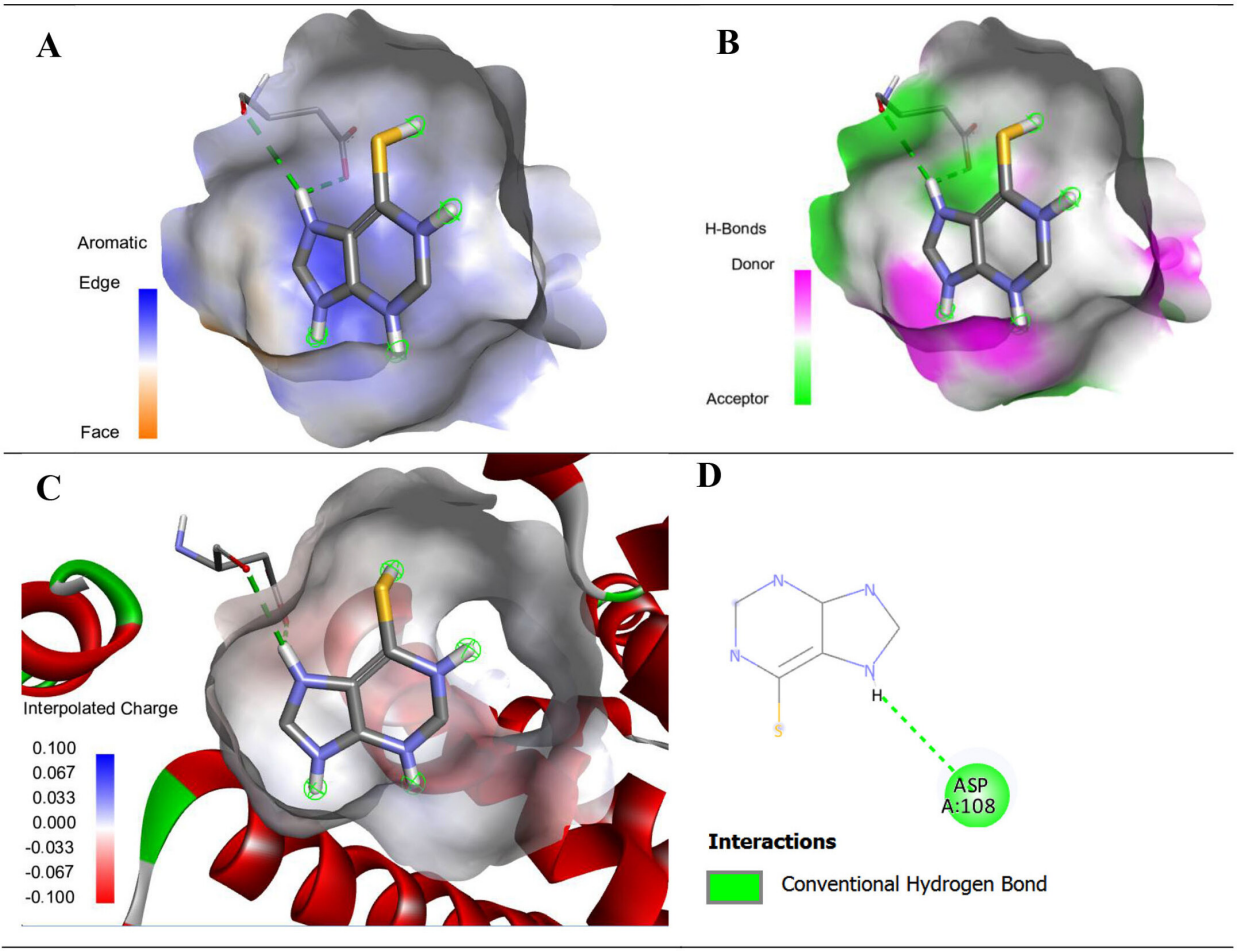
Aromatic (A), hydrogen bond (B), charge (C) and two-dimensional (D) interactions of the drug merceptopurine with the protein HSA (human serum albumin) PDB ID = 1e78 with

**Figure 12.**
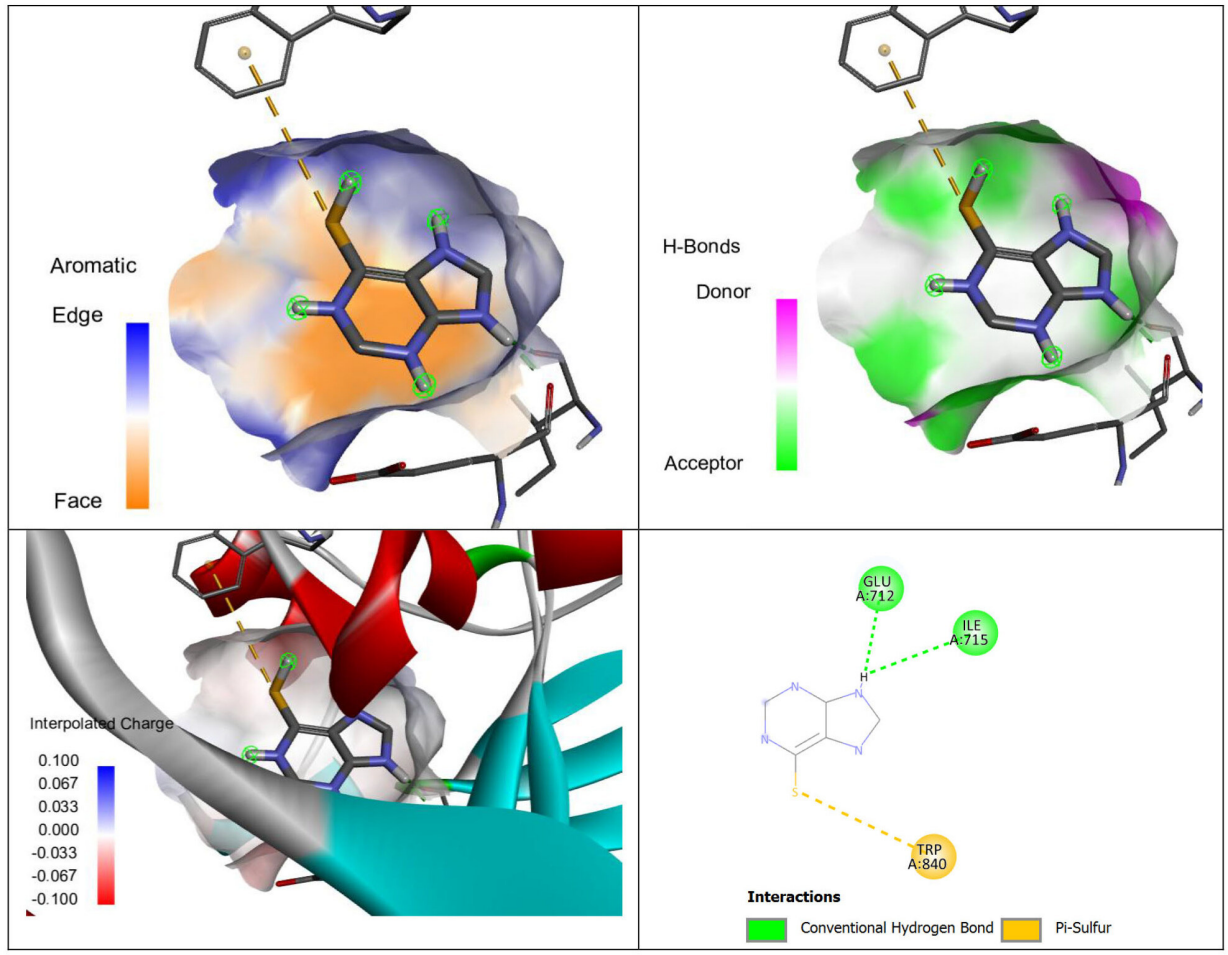
Aromatic (A), hydrogen bond (B), charge (C) and two-dimensional (D) interactions of the drug merceptopurine with the protein HGPRT PDB ID = 4fm9.

The detailed interactions between MP and the targets, HSA and HGPRT are given in table 8. In the case of HSA, the interactions present are conventional hydrogen bonds. The conventional hydrogen bonds ensure the strong binding between the ligand-protein complex, i.e. MP-HGA. In the case of HGPRT target, there are conventional hydrogen bonds, and pi-sulfur interactions. Again the larger number of interacting sites and relatively smaller distances between the interacting sites shows the MP to be more potent inhibitor against HGPRT. The visual illustration of the HSA and HGPRT interactions with MP are given in figures 11 and 12.

**Table 8.** The binding interactions of merceptopurine with protein HSA and HGPRT.

ligand	protein	amino acid	interactions	distance
merceptopurine	HSA	N:UNK1:H - A:ASP108:O	conventional hydrogen bond	2.8269
		N:UNK1:H - A:ASP108:OD1	conventional hydrogen bond	2.47297
	HGPRT	N:UNK1:H - A:GLU712:O	conventional hydrogen bond	1.95297
		N:UNK1:H - A:ILE715:O	conventional hydrogen bond	3.03552
		N:UNK1:S - A:TRP840	pi-sulfur	5.40525

## 9. pH effect

The release of drug from the carrier vehicle at the target site is one of the critical steps during the drug delivery process. The pH around the malignant cell environment is usually less than 6 as compared with normal healthy cells which have the pH in the range of 7.35−7.45 [47]. Therefore, the pH effect is also investigated on the studied drug-capsule complexes, i.e. AM@cap and MP@cap. We performed DFT simulations of complexes in an acidic media. For creating an acidic environment, we protonated the ends (O-atoms) of the cavitand-based nanocapsule and optimized the structures again at the same level of theory. The adsorption energies of AM@cap and MP@cap after protonation (acidic media) decrease drastically from −24.01 and −26.02 kcal mol^−1^ to –12.22 and −0.61 kcal mol^−1^, respectively. Overall, the decrease in adsorption energy indicates the easy release or off-loading of selected drugs from the nanocapsule at the target site. Moreover, the change or decrease in E_int_ is higher in the case of MP@cap. The reason can be overall more polar nature of the MP@cap complex as studied through its higher dipole moment. The more polar complex interacts with the polar environment strongly and overall loses the individual interactions, i.e. the interactions in between the drug (MP) and the nanocapsule.

## 10. Conclusions

Herein, the new drug delivery vehicle, i.e. the cavitand-based nanocapsule, has been employed for AM and MP drug molecules. The most stable orientation of AM and MP inside the nanocapsule shows interaction energy of −24.01 and −26.02 kcal mol^−1^, respectively. Moreover, the drug molecules and the nanocapsule interaction with each other with respect to charge transfer has been studied through NBO and EDD analysis, which shows greater charge transfer in the case of MP@cap as compared with AM@cap. Similarly, the FMOs analyses show the greater reduction of HOMO-LUMO gap when MP is encapsulated in nanocapsule (MP@cap) as compared with encapsulation of AM (AM@cap). Furthermore, the nature and strength of the non-covalent interactions involved in stabilizing drug molecules inside the nanocapsule’s cavity are evaluated through QTAIM and NCI analyses. The outcomes of both the NCI and QTAIM analyses show that the AM and MP are stabilized inside the nanocapsule mainly through hydrogen bonding and London dispersion forces. QTAIM analysis shows stronger interaction forces between MP and nanocapsule in the case of MP@cap complex as compared with AM@cap. Overall, the outcomes of the interaction energy, NBO, EDD, FMO, NCI, and QTAIM analyses can be correlated and nanocapsule acts as a better carrier for anti-cancer drug (MP). Additionally, dipole moment analysis results are also consistent with the other results, revealing that nanocapsule is able to release the drug (MP) on the target site more efficiently as compared with AM drug molecule. Additionally, we have employed AIMD analysis and molecular docking to find the dynamical stability of drug delivery system after drug loading and interaction of drug molecules with different targets to specify the exact targets of the selected drugs, respectively. We may conclude that cavitand-based nanocapsule shows better results when used as a carrier for MP anti-cancer drug molecule for drug delivery.

## Data Availability

All the supporting data is provided in the 'electronic supplementary material information' file [48].
